# Pernicious Anæmia: Its Pathology, Septic Origin, Symptoms, Diagnosis, and Treatment

**Published:** 1901-06

**Authors:** 


**Pernicious Anæmia: its Pathology, Septic Origin, Symptoms, Diagnosis, and Treatment.** By WILLIAM HUNTER, M.D. Pp. xvi., 464. London: Charles Griffin and Company, Limited. 1901.

The disease first described by Addison in 1855 under the name "Idiopathic Anæmia," and afterwards re-described in 1871 by Biermer under its present name, has been hitherto so wrapt in mystery as to justify the description given by Pye-Smith in 1883, who remarked: "The essence of this disease was, that it was without any symptoms during life, and without any lesions after death, which could not be explained as directly due to the anæmia."[1] The investigations now recorded have changed all this: they give the results of many years of pains-taking and laborious work, but this has not been unproductive, inasmuch as it has been possible to deduce conclusions of a very definite character which lead to important results. "As regards its *nature*, they show the disease to be not merely a special form of anæmia, but a definite and—in regard to mode of onset and site of infection—a well-characterised, chronic, infective disease, localised to the alimentary tract, one in which long-lasting *sepsis*, oral and gastric, plays an essential and important antecedent and concurrent part. As regards its *symptoms*, so far from the anæmia being the sole feature of the disease and the cause of all its symptoms and lesions, it is only one of the symptoms; and there are at least three other groups, hæmolytic, gastro-intestinal, and toxic—no less striking, in their grouping no less characteristic, and, in regard to the site of infection, far more instructive than the actual anæmia itself—caused, not by the anæmia itself, but by the infective agencies underlying the disease. As regards *diagnosis*, the disease can, even in its early stages, if not easily, yet with certainty, be diagnosed both during life and after death; *during life*, by having regard to (1) its mode of onset, with special reference to its oral and gastric symptoms; (2) the degree of blood change, out of all proportion to that caused by organic or wasting disease even when severe; (3) the characteristic groups of symptoms I have described; and *after death* by the no less characteristic changes

[1] *Guy's Hosp. Rep.*, 1883, xli. 219.

in the liver, bile, and kidneys. Lastly, as regards *treatment*, the conclusions suggest new lines in regard both to the prevention of the disease, and possibly also to its permanent arrest after it has commenced."

These remarkable results are difficult to realise; that we should be asked to accept the statement that the disease has a special (infective) etiology underlying it would be too much to ask if we did not feel sure that the author is a reliable investigator and is not likely to be mistaken in his inferences. His work refers only to the special form of anæmia differentiated by Addison, and it occurs apart from the usual causes or concomitants of the anæmic state. He does not recognise any primary or secondary forms of the disease or any association with all ordinary anæmia-producing factors. The disease is shewn to be "not only a clinical, but also a pathological entity; no mere aggravation of an ordinary anæmia, but a special and distinct variety," and the author formulates the following definition :—

"Pernicious anæmia is a chronic infective disease localised in the alimentary tract:

*Caused* by a definite infection of certain parts of the mucosa of the alimentary tract, chiefly of the stomach, occasionally also of the mouth, and of the intestine.

*Characterised* by:

(1) Intermittent destruction of blood and increasing anæmia —and all the other pathological and clinical changes consecutive to these; *eg*., anæmia, lemon colour, urobilinuria, hemorrhages, dyspnœa, palpitation, œdema,—as the result of the hæmolytic action of poisons in the blood.

(2) Periodic disturbance of the alimentary tract, chiefly of the stomach and the intestine, as local irritant effects of the infection on the alimentary canal; and

(3) Occasional toxæmic attacks, characterised by fever, sweating, general nervous symptoms, not infrequently by effects; *e.g*., numbness, tingling, ataxia, absence of reflexes, denoting deeper nervous changes, such as peripheral neuritis, sclerosis of the cord.

As regards treatment, the objects to be aimed at are three:

1. Prevention and removal of the infective cause.

2. Removal of the gastro-intestinal conditions favouring its operation.

3. To combat the chief symptoms."

The hygiene of the mouth demands, therefore, the most scrupulous attention. Further, it is needful to remove or lessen the septic catarrh of the stomach. The antiseptics which the author appears to prefer are salicylic acid and salicylate of bismuth. These measures should not replace, but should be supplemented by, the use of arsenic. If they fail treatment by antistreptococcic serum holds out some prospect of improvement. We hope that this prospect will in due time be verified, and that the work of Dr. Hunter will direct the attention of medical observers generally to cases which have been frequently overlooked until they are beyond the reach of any kind of treatment.

